# The Research on a Collaborative Management Model for Multi-Source Heterogeneous Data Based on OPC Communication

**DOI:** 10.3390/s25247517

**Published:** 2025-12-10

**Authors:** Jiashen Tian, Cheng Shang, Tianfei Ren, Zhan Li, Eming Zhang, Jing Yang, Mingjun He

**Affiliations:** School of Mechanical Engineering, Hebei University of Science and Technology, Shijiazhuang 050018, China

**Keywords:** multi-source heterogeneous data, edge-centric computing framework, hybrid OPC communication stack, dynamic time warping (DTW), machine-learning-driven dynamic scheduling

## Abstract

Effectively managing multi-source heterogeneous data remains a critical challenge in distributed cyber-physical systems (CPS). To address this, we present a novel and edge-centric computing framework integrating four key technological innovations. Firstly, a hybrid OPC communication stack seamlessly combines Client/Server, Publish/Subscribe, and P2P paradigms, enabling scalable interoperability across devices, edge nodes, and the cloud. Secondly, an event-triggered adaptive Kalman filter is introduced; it incorporates online noise-covariance estimation and multi-threshold triggering mechanisms. This approach significantly reduces state-estimation error by 46.7% and computational load by 41% compared to conventional fixed-rate sampling. Thirdly, temporal asynchrony among edge sensors is resolved by a Dynamic Time Warping (DTW)-based data-fusion module, which employs optimization constrained by Mahalanobis distance. Ultimately, a content-aware deterministic message queue data distribution mechanism is designed to ensure an end-to-end latency of less than 10 ms for critical control commands. This mechanism, which utilizes a “rules first” scheduling strategy and a dynamic resource allocation mechanism, guarantees low latency for key instructions even under the response loads of multiple data messages. The core contribution of this study is the proposal and empirical validation of an architecture co-design methodology aimed at ultra-high-performance industrial systems. This approach moves beyond the conventional paradigm of independently optimizing individual components, and instead prioritizes system-level synergy as the foundation for performance enhancement. Experimental evaluations were conducted under industrial-grade workloads, which involve over 100 heterogeneous data sources. These evaluations reveal that systems designed with this methodology can simultaneously achieve millimeter-level accuracy in field data acquisition and millisecond-level latency in the execution of critical control commands. These results highlight a promising pathway toward the development of real-time intelligent systems capable of meeting the stringent demands of next-generation industrial applications, and demonstrate immediate applicability in smart manufacturing domains.

## 1. Introduction

Pivotal for modern industrial production, the deep integration and efficient convergence between information and communication technology (ICT) and industrial data rest upon three foundational pillars: digitalization, networking, and intelligence [1]. This imperative is underscored by China’s 14th Five-Year Plan, which prioritizes the advancement of high-end, intelligent manufacturing [2]. Specifically, the Plan actively fosters the deep integration of the Internet and big data across diverse industrial sectors, championing innovation-driven growth. It advocates for collaborative data management practices and facilitates the exploitation of multi-source data resources, thereby underpinning sustained economic expansion.

The Industry 4.0 and IoT ecosystems encompass diverse data sources including widely distributed and heterogeneous sensors, control systems, and monitoring devices across multiple subsystems, posing significant challenges for data management [3]. These challenges are intensified by the proliferation of heterogeneous data streams and edge devices, as well as the coexistence of high-frequency real-time data streams and long-interval data acquisition cycles [4]. Compounding these challenges, the intricate nature of multi-source heterogeneous data and persistent interoperability issues expose critical limitations that are inherent in traditional manual operations. Such limitations manifest as inefficient data collection and processing, compromised data quality, and inadequate standardization. However, the effective processing of real-time data presents persistent challenges within resource-constrained environments. These challenges critically undermine both data synergy in manufacturing contexts and efficient resource utilization. Consequently, they present fundamental obstacles for the advancement of future computing systems.

**Figure 1:**
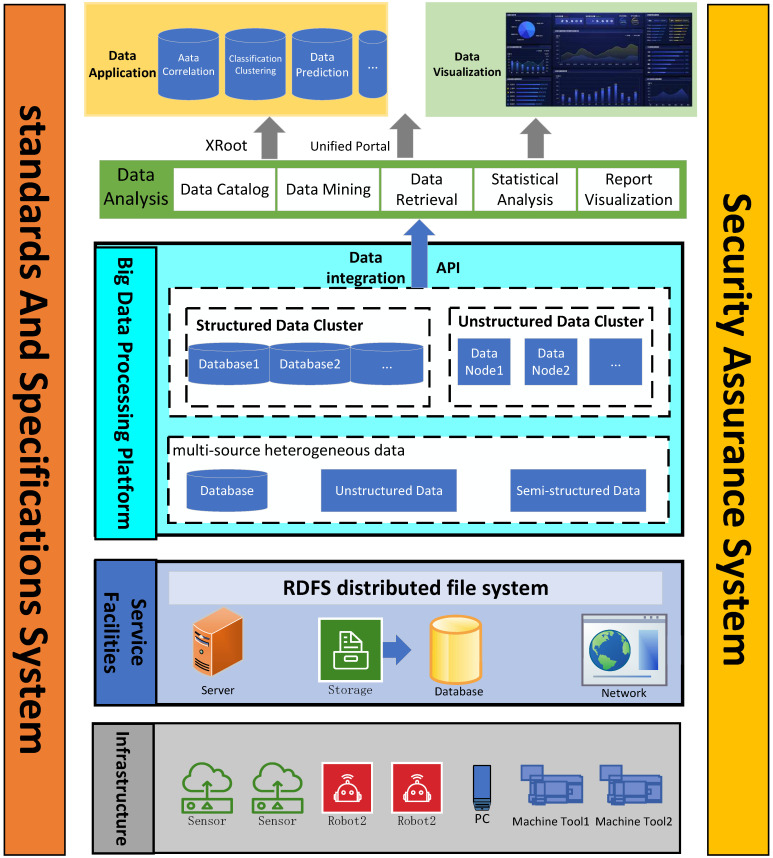
Overview of Multi-Source Heterogeneous Data.

The multi-source heterogeneous nature of industrial data manifests primarily in the diversity of data sources and the complexity of data types, as illustrated in Figure 1. Significant disparities exist across multiple dimensions, including data sources, data types, collection methods, formats, update frequencies, storage structures, and semantic definitions [5]. These variations thereby complicate data fusion processes. Furthermore, time asynchrony among different data sources exacerbates the challenge, negatively impacting the accuracy of coordinated analysis and decision-making [6].

Existing solutions based on single paradigms, such as OPC UA or MQTT, exhibit limitations in jointly optimizing communication, computation, and control (3C). Although OPC UA offers superior information modeling capabilities that ensure reliable command delivery, its centralized architecture constrains data distribution efficiency [7]. Conversely, MQTT offers superior performance in lightweight data broadcasting but lacks support for robust real-time control and semantic interoperability. Consequently, a hybrid architecture that effectively integrates their complementary strengths becomes essential [7].

Existing approaches to addressing these challenges are frequently limited by their inability to jointly optimize communication, computation, and control (3C). This critical gap necessitates focused exploration in three key areas: (1) a unified modeling framework, (2) collaborative integration strategies, and (3) intelligent processing methodologies specifically designed for multi-source heterogeneous industrial data. Ultimately, this research will provide the foundational architecture necessary for the coordinated advancement of manufacturing intelligence and industrial Internet ecosystems. It will further enable the systematic convergence of cross-domain data governance, distributed computing optimization, and knowledge-driven decision support systems.

## 2. Hybrid Data System Architecture and Communication Protocol Stack Design

Widely recognized as an open, cross-platform communication standard, OPC technology has achieved extensive adoption across industrial applications. Its core strengths lie in enabling real-time data exchange and unified management [8]. This interoperability capability proves crucial for integrating industrial robots, sensors, and domain-specific information models, effectively resolving challenges associated with data interoperability and semantic integrity [9]. Leading equipment manufacturers, including Siemens and ABB, inherently support the OPC protocol [7]. For devices lacking inherent OPC compatibility, protocol conversion gateways provide seamless interconnection solutions, further underscoring OPC’s maturity and significant value in equipment integration [10].

### 2.1. Design Principles of Hybrid Data System Architecture and Communication Protocol Stack

Central to this hybrid architecture’s design is the adoption of a mixed communication interaction mode. This strategy specifically addresses the diverse requirements of industrial systems, encompassing both low-latency real-time control and efficient, reliable global state management. The distinct characteristics of the OPC Client/Server [11], OPC Pub/Sub [12,13], and P2P [14,15] communication modes underpinning this approach are delineated in Table 1.

### 2.2. Hybrid Architecture of Data Systems and Communication Protocol Stack Design Scheme

**Figure 2:**
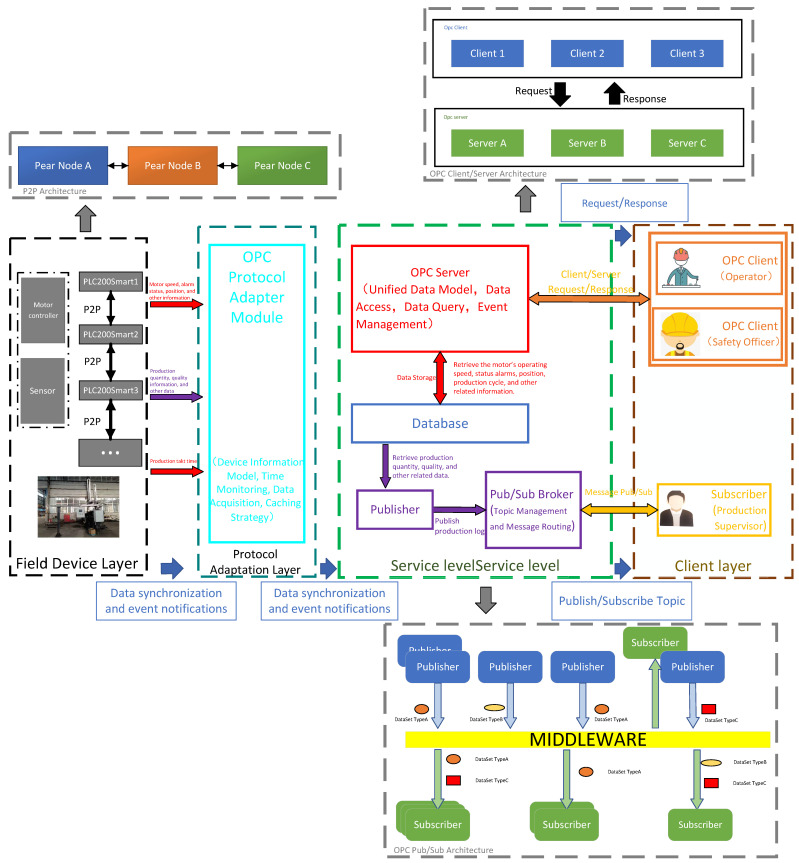
Hybrid Architecture Design.

**Figure 3:**
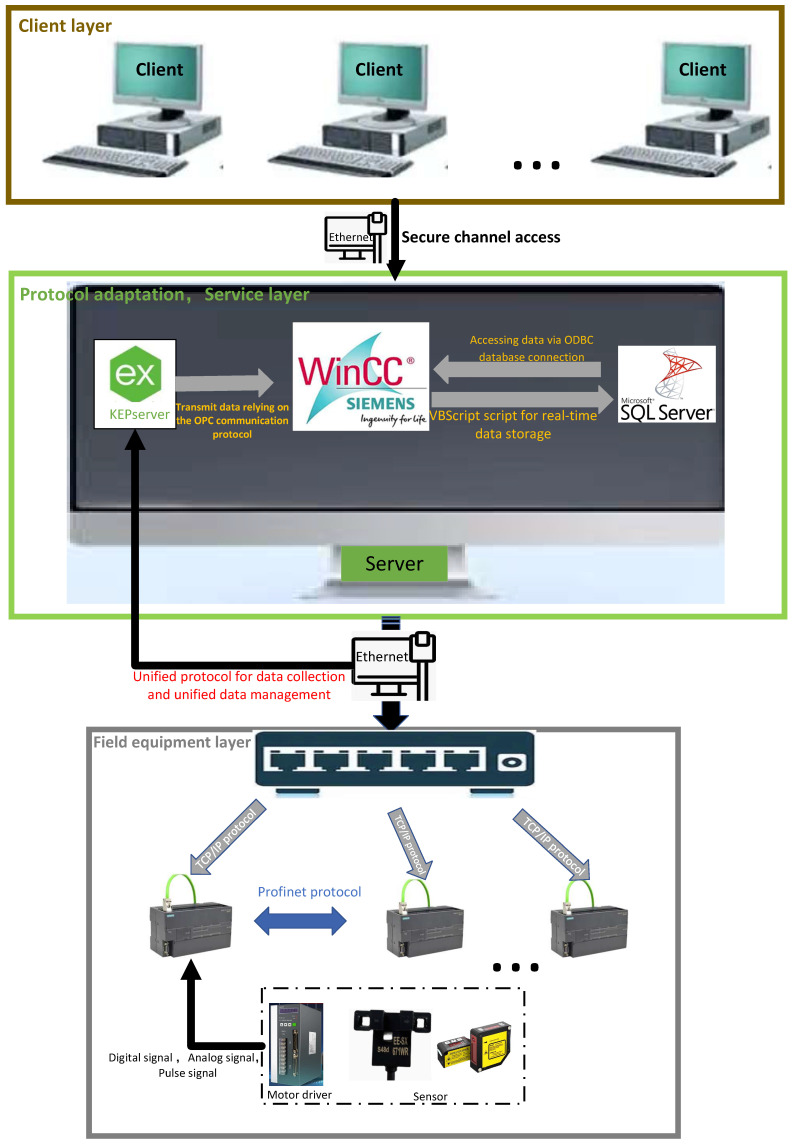
Communication Protocol Stack Design.

Figure 2 and Figure 3 jointly depict the interplay between the data-system architecture and its underlying communication stack. The data-system architecture supplies the structural blueprint, while the underlying communication stack translates this blueprint into executable protocol primitives. Their synergistic coupling is pivotal for guaranteeing efficiency, reliability, and security across the entire data pipeline [16].

Architecturally, the system is engineered to offer end-to-end functionality, encompassing field-level data acquisition, heterogeneous-protocol-based mediation, and centralized governance. A data-type-driven publish-subscribe mechanism coordinates distributed components under real-time constraints, meeting multi-tenant monitoring requirements in complex industrial environments. Complementarily, the protocol stack supports this architecture through unified encoding schemes and secure channels, enabling low-latency data acquisition and scalable multi-client monitoring [17].

Device Layer: Drivers, sensors, and the PLC-200 Smart form a resilient PROFINET mesh supporting peer-to-peer (P2P) reconfiguration and dynamic connectivity. These components connect upstream to the supervisory host via Ethernet-switched TCP/IP infrastructure.

Service Layer: The host system interfaces with field devices through dedicated Ethernet connections, utilizing WinCC for data acquisition, distribution, visualization, and alarm management. Data persistence is maintained through ODBC-mediated SQL Server backend database, whereas real-time monitoring and business logic are handled by an OPC server. A dedicated Pub/Sub broker ensures low-latency distribution of operational data.

Client Layer: The system provides concurrent, role-aware access for operators, safety officers, and production supervisors, delivering context-specific data streams to each user group without interference.

## 3. Intelligent Data Management System

### 3.1. Event-Triggered Adaptive Sampling Mechanism Based on Improved Kalman Filter Dynamic Adjustment

#### 3.1.1. Event-Triggered Adaptive Sampling Mechanism

**Figure 4:**
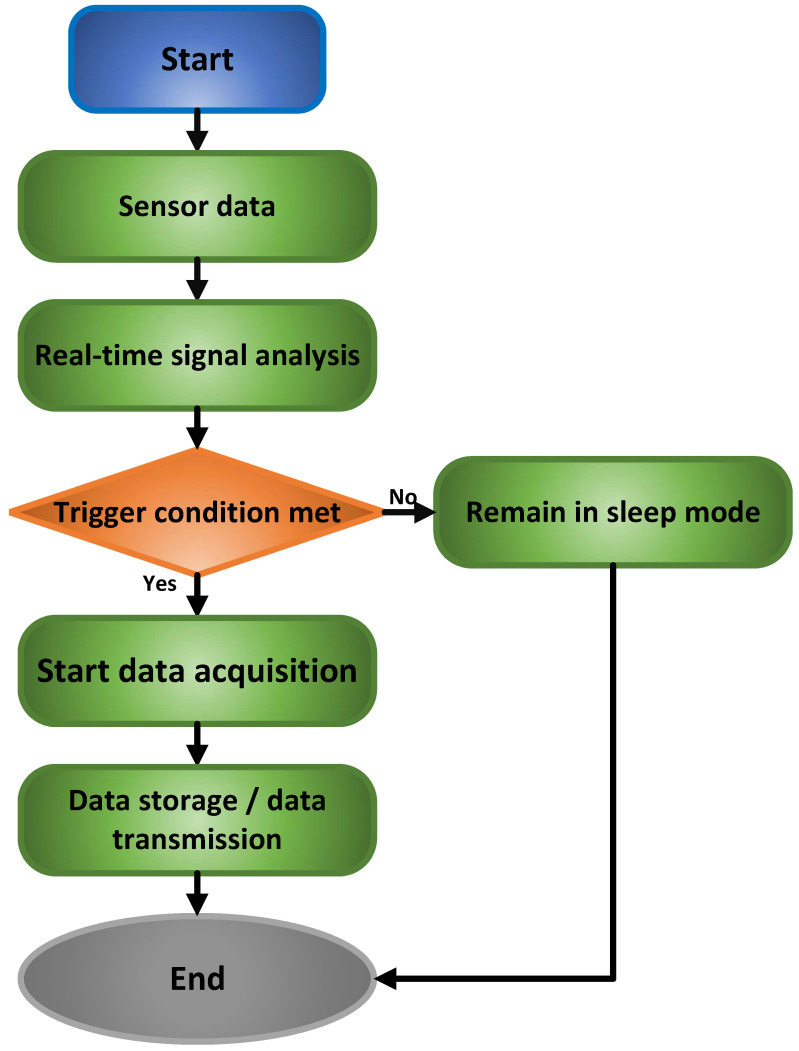
Event-Triggered Data Acquisition Process.

The system employs an intelligent triggering mechanism with multi-level states and composite conditions, and evaluates trigger conditions using composite logic. This logic integrates key signal features, including amplitude, rate of change, and duration. When these trigger conditions are not satisfied, the system enters a low-power sleep mode, preserving only essential monitoring functions. Trigger activation, however, initiates high-speed data acquisition, capturing comprehensive signal waveforms both preceding and following the event. Subsequently, the acquired data undergo timestamping, format conversion, and transmission to the database via a dedicated interface, thus completing the acquisition cycle. This entire process is interrupt-driven and managed by a state machine, guaranteeing strictly controlled response delays and efficient module coordination [18,19]. Figure 4 illustrates this event-triggered data acquisition process.

#### 3.1.2. Improved Kalman Filter Dynamic Adjustment Algorithm

(1) Core Model of Kalman Filter Dynamic Adjustment Algorithm

The system’s data acquisition functionality is implemented within an adaptive sampling frequency framework based on an improved Kalman filter algorithm. This method optimizes the sampling rate in real-time through recursive estimation of state errors, leveraging sensor fusion of encoder-measured motor rotational velocity and mechanical transmission dynamics [20,21]. The system establishes a kinematic state-space model by defining the end-effector position Xk and motor rotational velocity Vk, as composite state variables ${\hat{x}}_{k}={\left[y_{k},v_{k}\right]}^{T}$. The mechanical transmission relationship between these variables is encapsulated in the state transition matrix A, while the control input matrix B maps the motor drive voltage $u_{k}$ to the state space. Given the posterior state estimate ${\hat{x}}_{k-1}$ from the previous time step k − 1, the Kalman filter performs two-stage processing: (1) a priori state prediction ${\hat{x}}_{k}^{-}=A{\hat{x}}_{k-1}+Bu_{k}$, and (2) measurement update incorporating encoder feedback, enabling closed-loop optimization of sampling frequency based on residual error thresholds.

(1) $$A=\left[\begin{array}{cc} 1 & \Delta t\\ 0 & 1 \end{array}\right]$$

(2) $$B=\left[\begin{array}{c} \Delta t\\ 0 \end{array}\right]$$

The prior error covariance matrix $P_{k}^{-}$ is computed synchronously during the Kalman filter’s prediction phase to quantify state estimation uncertainty.

(3) $$P_{k}^{-}=AP_{k-1}A^{T}+Q$$

where $P_{k}^{-}$ denotes the prior state estimation uncertainty. Q=diag([0.01,0.1]) is the process noise covariance matrix, which reflects the uncertainty in the motor dynamics model.

After acquiring the measurement $z_{k}$ the system calculates the Kalman gain matrix:

(4) $$K_{k}=P_{k}^{-}H^{T}{\left(HP_{k}^{-}H^{T}+R\right)}^{-1}$$

In the equation, *H* is the observation matrix. *R* is the measurement noise covariance matrix, and $K_{k}$ determines the contribution weight of the observed value to the state update. The state estimation correction process is completed via the following equation:

(5) $${\hat{x}}_{k}={\hat{x}}_{k}^{-}+K_{k}\left(z_{k}-H{\hat{x}}_{k}^{-}\right)$$

With $z_{k}$ representing encoder-measured position/speed values corrupted by Gaussian noise. The posterior error covariance is updated:

(6) $$P_{k}=\left(I-K_{k}H\right)P_{k}^{-}$$

where *I* denotes the identity matrix.

(2) Enhance robustness to outliers

Motor encoders are prone to electromagnetic interference and mechanical vibrations, inducing measurement outliers. Traditional Mean Squared Error (MSE) is highly sensitive to such outliers, causing abnormal increases in the normalized estimation error Δx. This may prematurely trigger high-frequency sampling. To address this, the system incorporates the Huber loss function into Δx calculation, significantly suppressing encoder outlier impacts.

(7) $$\Delta x=\sqrt{{\left(z_{k}-H{\hat{x}}_{k}^{-}\right)}^{T}P_{k}^{-1}\left(z_{k}-H{\hat{x}}_{k}^{-}\right)}$$

Truncation threshold $c=1.5\sqrt{R}$. When $|z_{k}-H{\hat{x}}_{k}^{-}|>c$, adopt a linear loss function to reduce the impact of outliers. The Huber loss function introduces a threshold mechanism that preserves estimation accuracy by applying quadratic loss to small errors while reducing the influence of outliers through linear loss for larger deviations. This conditional loss-allocation strategy significantly enhances the algorithm’s robustness against occasional sensor anomalies.

(3) Introduce a multi-condition triggering mechanism

Sole reliance on normalized error Δx as the single triggering condition faces limitations in complex operational scenarios. For example, during abrupt control command changes (e.g., emergency braking), the system requires an immediate response. However, computational delays inherent in error calculation hinder timely reactions. Moreover, external disturbances like mechanical shocks may cause covariance divergence, which degrades estimation accuracy and necessitates temporary increases in the sampling rate for the system. Therefore, supplementary triggering conditions beyond Δx are implemented:

(a) Emergency command detection:

(8) $$∥u_{k}-u_{k-1}∥>0.2u_{\max}$$

The system activates maximum sampling rate $f_{\max}$.

(b) Covariance surge detection:

(9) $$\mathrm{Tr}\left(P_{k}\right)>1.5\cdot \mathrm{Tr}\left(P_{k-1}\right)$$

The mechanism triggers temporary high-frequency sampling to prevent estimation failure.

To avoid abrupt rate transitions, smooth frequency adjustment based on error gradients is adopted. A nonlinear mapping function dynamically computes adjustment coefficients according to Δx’s deviation from thresholds:

(10) $$f_{k+1}=\left\{\begin{array}{cc} f_{k}\cdot \exp\left(\gamma (\Delta x-\alpha_{k})\right) & \Delta x>\alpha_{k}\\ f_{k}\cdot \exp\left(-\eta (\beta_{k}-\Delta x)\right) & \Delta x<\beta_{k} \end{array}\right.$$

where, parameters: γ,η are the attenuation factors.

(4) Introduction of Dynamic Thresholds and Adaptive Adjustment Strategies

Fixed thresholds (α,β) exhibit limited adaptability to dynamic motor operating states with uncertainties, potentially causing false high-frequency triggering and insensitivity issues. To address this, real-time adjustable thresholds are introduced, which are dynamically tuned by the system based on the trace of the historical error covariance matrix ($\mathrm{Tr}(P_{k})$):

(11) $$\begin{array}{cc} \alpha_{k} & =1.2\cdot \sqrt{\mathrm{Tr}(P_{k}^{-})}\\ \beta_{k} & =0.8\cdot \sqrt{\mathrm{Tr}(P_{k}^{-})} \end{array}$$

During motor acceleration, increased model uncertainty elevates ($P_{k}^{-}$), thereby relaxing $\alpha_{k}$ to avoid excessive high-frequency sampling. Conversely, during steady-state operation, decreased $P_{k}^{-}$ tightens $\alpha_{k}$, enabling prompt sampling rate reduction. This mechanism operates analogously to a feedback control system, dynamically adjusting the activation threshold based on real-time variations in system operating states.

(5) Introducing online estimation of noise covariance matrix

During motor operation, both process noise (*Q*) and observation noise (*R*) exhibit non-stationary statistical characteristics. When their time-varying properties deviate from initial assumptions, the fixed noise covariance framework induces model-plant mismatch, degrading Kalman filter performance through suboptimal state estimation. Furthermore, prolonged system operation under static covariance parameters exacerbates this degradation via error propagation mechanisms, leading to cumulative divergence in state estimates and ultimately causing the model to diverge from the true system. To address these limitations, we propose an adaptive noise covariance recalibration approach leveraging a forgetting factor (λ). This method enables real-time dynamic updates of process noise covariance ($Q_{k}$) and observation noise covariance ($R_{k}$) by exponentially weighting historical measurement residuals, mathematically formulated as follows:

(12) $$\begin{array}{cc} Q_{k} & =\lambda Q_{k-1}+\left(1-\lambda \right)K_{k}z_{k}z_{k}^{T}K_{k}^{T}\\ R_{k} & =\lambda R_{k-1}+\left(1-\lambda \right)\left(z_{k}-H{\hat{x}}_{k}\right){\left(z_{k}-H{\hat{x}}_{k}\right)}^{T} \end{array}$$

The forgetting factor λ (ranging from 0.95 to 0.99) serves as a critical parameter in the adaptive recalibration of process noise covariance $Q_{k}$ and observation noise covariance $R_{k}$. The value of λ determines the algorithm’s memory length concerning past data. A larger value of λ results in a smoother response to current noise variations, thereby enhancing the algorithm’s immunity to transient disturbances. During prolonged operation, the rise in motor temperature induces thermal drift in electromagnetic parameters, which include variations in copper resistance and magnetic core saturation. In turn, these variations give rise to time-varying electromagnetic noise. This non-stationary noise compromises the statistical consistency of the Kalman filter’s noise model, leading to suboptimal gain computation and error covariance inflation. By dynamically modulating the exponential decay rate of historical data influence, λ enhances the filter’s robustness against time-varying noise, thereby effectively reducing estimation errors originating from model mismatch.

A larger value of λ results in a smoother response to current noise variations, thereby enhancing the algorithm’s immunity to transient disturbances. To mitigate the latency inherent in delivering sampling-rate adjustment commands to the hardware, a state-prediction model is introduced to compensate for this delay. By forecasting the system state for the next time step, the model enables prompt and accurate updates to the sampling rate, ensuring that control actions remain timely and effective.

(13) $${\hat{x}}_{k+1|k}=A{\hat{x}}_{k}+Bu_{k}$$

Table 2 summarizes the symbols of the improved Kalman filter algorithm and their corresponding parameter definitions.

**Figure 5:**
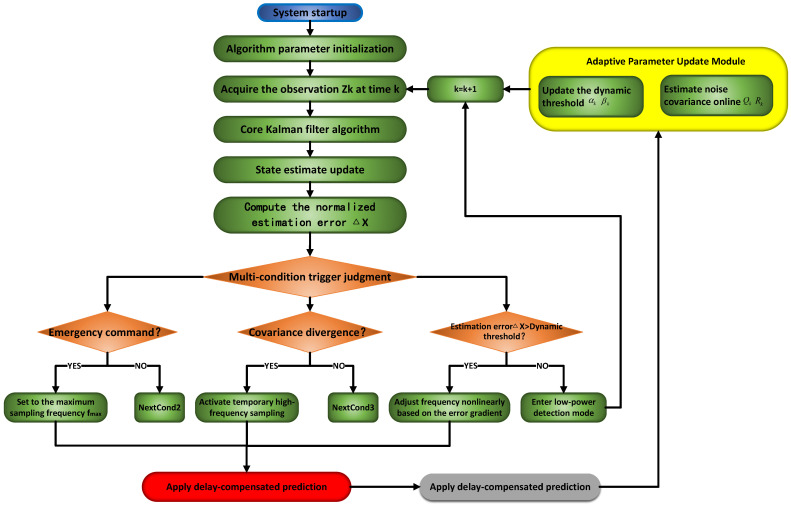
Overall workflow of the event-triggered adaptive data-acquisition mechanism based on the improved Kalman filter algorithm.

Figure 5 illustrates the overall workflow of the event-triggered adaptive data acquisition mechanism implemented in this data system, which is built upon the enhanced Kalman filter algorithm.

### 3.2. Heterogeneous Data Fusion Based on the Dynamic Time Warping (DTW) Algorithm

Multi-source heterogeneous data systems inherently involve asynchronous sampling from diverse devices and sensors operating at non-uniform frequencies. To address synchronization challenges in such environments, this study employs the Dynamic Time Warping (DTW) algorithm for temporal alignment of multi-modal sensor data streams [22,23,24,25].

Consider two rotary encoders measuring motor rotational velocity at distinct sampling frequencies $f_{1}$ and $f_{2}$ generating time series $S_{1}=\left\{s_{1}\left(t_{1}\right),\ldots ,s_{1}\left(t_{n}\right)\right\}$ and $S_{2}=\left\{s_{2}\left(t_{1}^{\prime }\right),\ldots ,s_{2}\left(t_{m}^{\prime }\right)\right\}$. The algorithm first constructs an n×m cost matrix *C*.

(14) $$c_{ij}=\underset{\mathrm{SpeedMatchingTerm}}{\underset{︸}{\frac{|v_{x}\left(t_{i}\right)-\alpha v_{y}\left(t_{j}^{\prime }\right)|}{\sigma_{X}}}}+\lambda \underset{\mathrm{AccelerationMatchingTerm}}{\underset{︸}{{\left|\frac{dv_{x}}{dt}\right|}_{t_{i}}-{\left.\frac{dv_{y}}{dt}\right|}_{t_{j}^{\prime }}}}\;$$

where α is the conversion coefficient from rotational speed to linear speed; $\sigma_{X}$ is the standard deviation of the velocity along the X-axis, which is utilized for normalization; λ regulates the weight proportion of the acceleration term; and $c_{ij}$ denotes the difference between the observations at time points $t_{i}$ and $t_{j}^{\prime }$. The accumulated cost matrix *D* is computed recursively using dynamic programming.

(15) $$D\left(i,j\right)=c_{ij}+\min\left\{\begin{array}{cc} D\left(i-1,j\right)+0.5c_{i-1,j} & X\text{-}\mathrm{axis}\;\mathrm{continous}\;\mathrm{movement}\;\mathrm{priority}\\ D\left(i,j-1\right)+0.3c_{i,j-1} & Y\text{-}\mathrm{axis}\;\mathrm{continous}\;\mathrm{movement}\;\mathrm{priority}\\ D(i-1,j-1) & \end{array}\right.$$

The algorithm sets adaptive window w(i)=|2+0.1i|, allowing the path to expand the search range during the acceleration phase. For d-dimensional feature data, the distance metric used is the Mahalanobis distance [26].

(16) $$\left|\right|s_{1}-s_{2}{\left|\right|}_{M}^{2}=\left(s_{1}-s_{2}\right)\wedge T\Sigma^{-1}\left(s_{1}-s_{2}\right)$$

Here, Σ represents the covariance matrix of the features [27], which effectively eliminates the influence of different scales and correlations among features [28].

Dynamic Time Warping (DTW) constructs a cost matrix by aligning two velocity sequences through time-axis warping.

Table 3 presents the notations and corresponding definitions for the DTW algorithm.

### 3.3. Content Routing-Aware Strategy-Based Message Queue Optimized Data Distribution Mechanism

To ensure stable low-latency performance of the message queue under dynamic loads, this system adopts a hybrid scheduling strategy characterized as “rules first”. The core of this strategy is a robust, priority-based rule scheduler designed to guarantee bounded latency for critical commands. Complementing this foundation, a lightweight non-core resource allocation prediction model is introduced to dynamically fine-tune the system resource parameters, thereby enhancing overall resource utilization efficiency. The scheduling mechanism is anchored by a deterministic rule engine that strictly enforces the following principles: high-priority queues (e.g., control commands) are assigned pre-allocated computational resources and possess preemption rights, while low-priority queues are processed only when all high-priority queues are empty.

**Figure 6:**
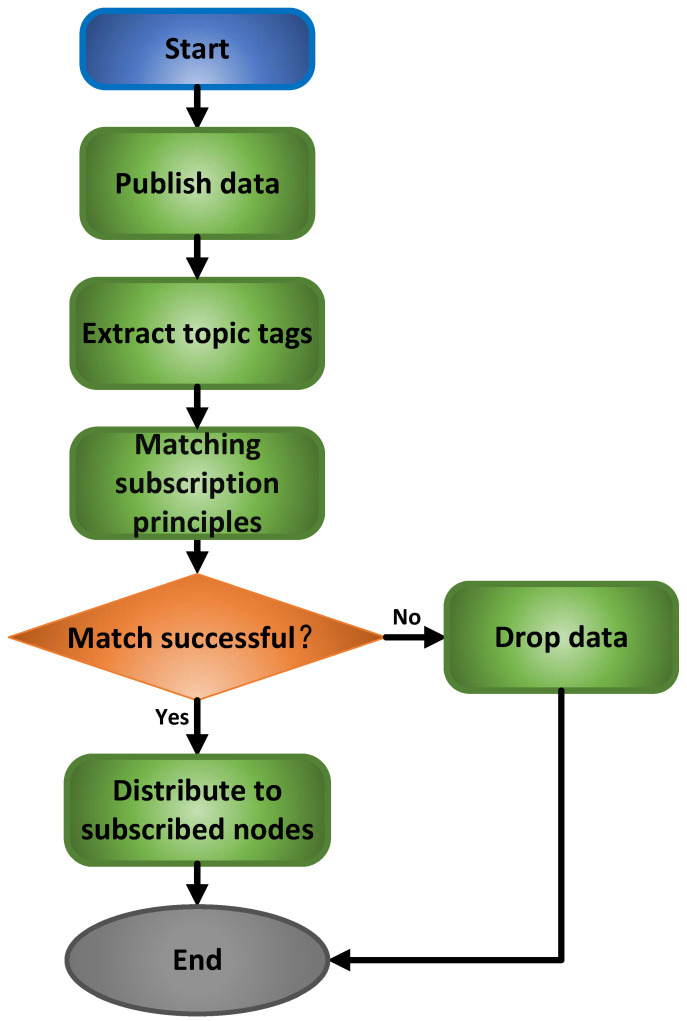
Content-aware Routing Strategy.

**Figure 7:**
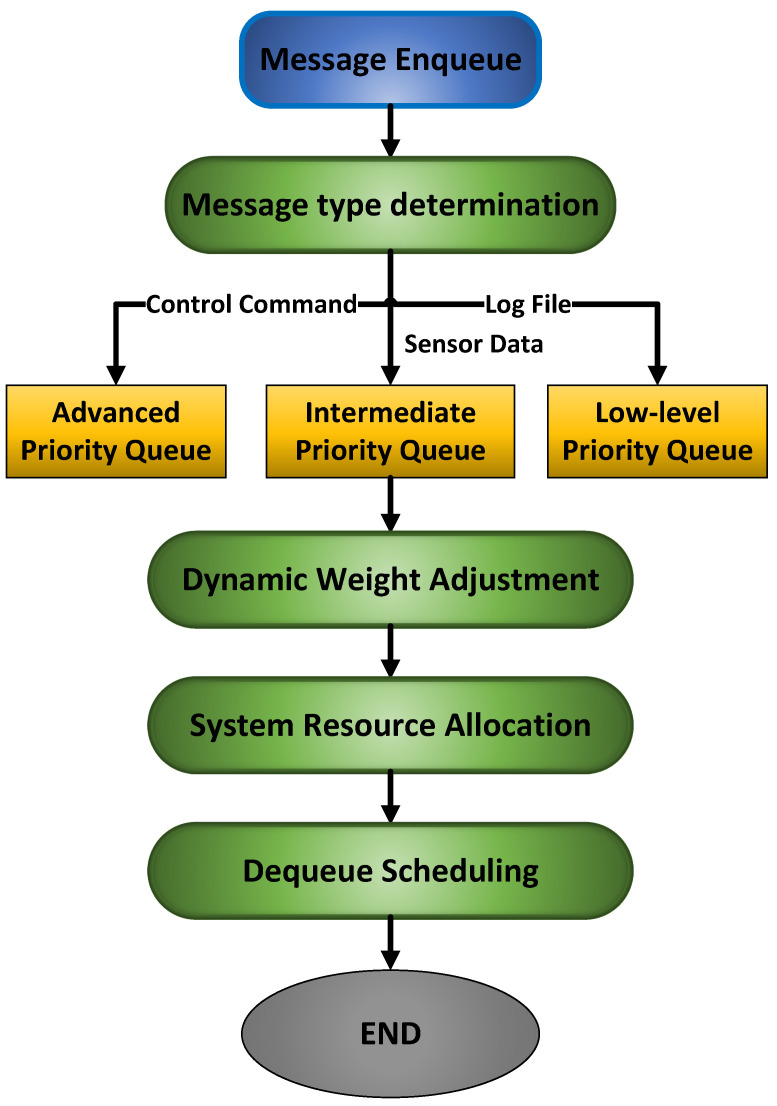
Message Queue Optimization Flow.

Guided by these design principles, the system first constructs an efficient content routing and message queue framework. As illustrated in Figure 6, the publisher node classifies and encapsulates data according to predefined topic tags, such as robot ID and task type. References [29,30,31] provide relevant information. Subsequently, a three-level message queue architecture (Figure 7) is implemented to support differentiated Quality of Service (QoS) levels [32,33]. To mitigate potential resource allocation redundancy resulting from static rules, a lightweight non-core prediction model is introduced. This model does not participate in critical scheduling decisions; instead, it dynamically forecasts whether the system is in an idle or busy state and adjusts resource weights for medium and low-priority queues accordingly. Inputs to the model consist of easily obtainable system-wide metrics, including the average length of high-priority queues, short-term historical data of overall system CPU utilization, and its variation trend. The output is a straightforward resource allocation weight. When the system is predicted to be in “idle” state, the model allows for greater resource usage by non-critical tasks; and under “busy” conditions, it reduces resource weights for non-critical tasks to preserve processing capacity for core operations.

The core of this architecture is a high-performance, deterministic rule-based scheduler. To ensure architectural extensibility for future performance tuning, the scheduler provides a standardized machine learning optimization interface. This interface is meticulously designed as a forward-looking feature to enable seamless integration of well-validated machine learning models with minimal adaptation effort. Crucially, any future ML-based strategies will not handle critical scheduling decisions, which are and will remain exclusively handled by the deterministic rule engine. This design fundamentally preserves the integrity and predictability of the system’s core architecture.

After classification and parsing, messages are dispatched to corresponding priority queues. The control command queue employs a preemptive memory pre-allocation mechanism, achieving zero-wait enqueueing. The dynamic scheduling module with closed-loop feedback automatically balances service loads and performs graceful degradation during system overloads, ensuring that the end-to-end latency of critical control commands remains consistently below 10 ms.

## 4. Experimental Verification

### 4.1. Experiment on Engineering Execution Mechanism and Upper Computer Configuration

**Figure 8:**
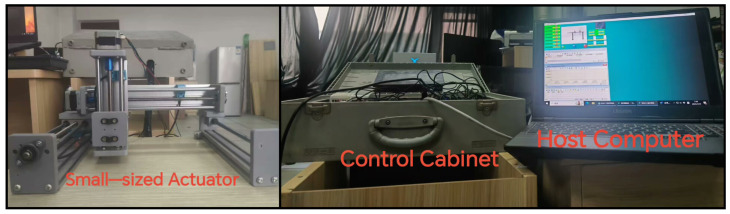
Small-Scale Gantry Robot Data Management Experiment.

As depicted in Figure 8, the experimental platform is hierarchically organized around three core components: (i) a miniature three-axis gantry robot that functions as the primary motion actuator, (ii) a Siemens S7-200 Smart PLC responsible for low-level trajectory execution, and (iii) a supervisory host computer that orchestrates high-level control strategies. Through tightly coupling the PLC with the host computer, the system is able to replicate, at reduced scale, the dynamic behaviors characteristic of full-sized gantry systems, thereby providing a controllable yet representative testbed for subsequent validation studies.

**Figure 9:**
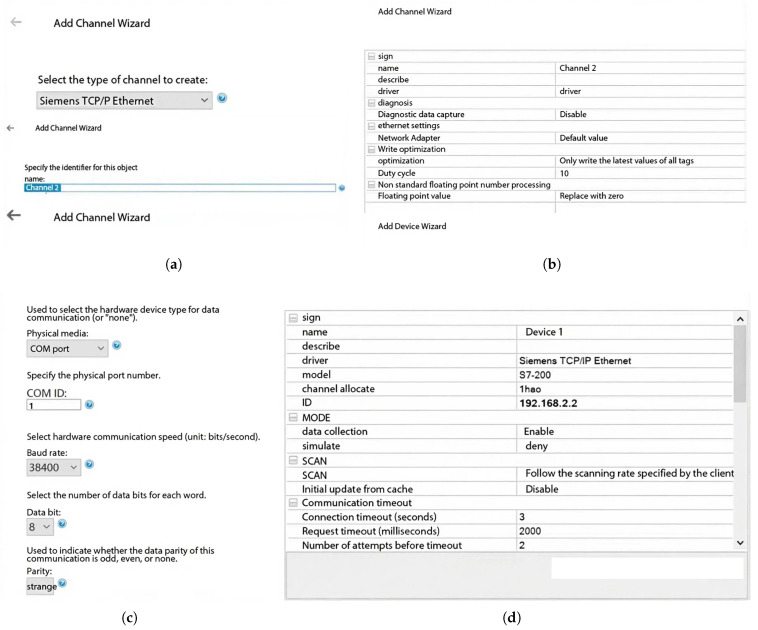
OPC Server Configuration Steps. (**a**) Configure channel type and naming; **(b**) Channel parameter attributes; (**c**) Server sampling and scanning par ameter configuration; (**d**) Connect device network properties.

**Figure 10:**
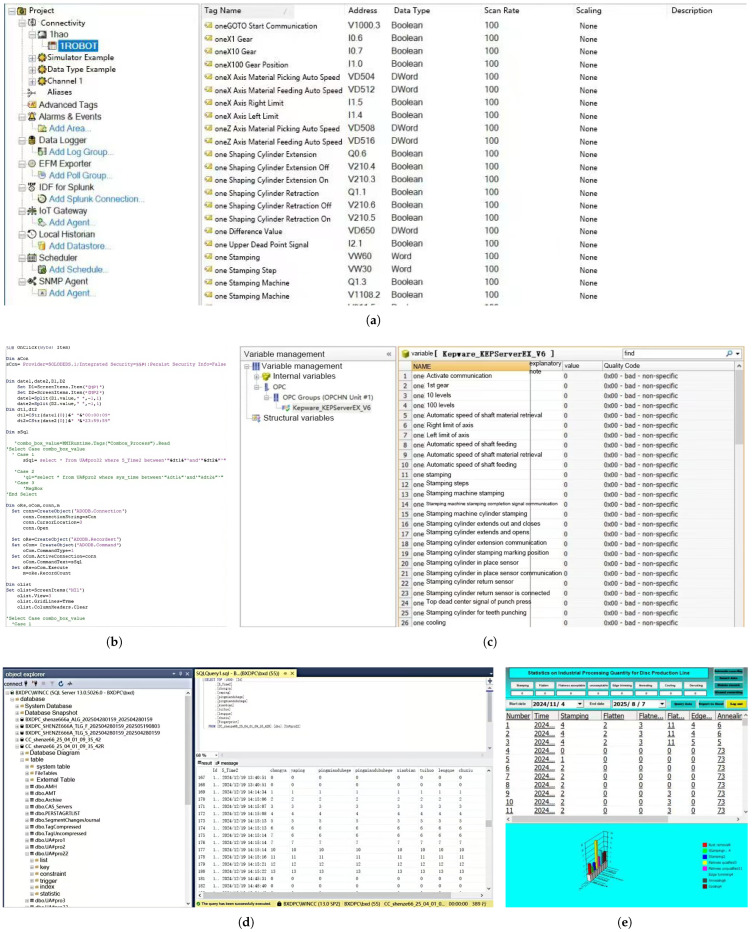
Server and Database Integration Overview. (**a**) KEPServer OPC Server Configuration; (**b**) Example of VB Script for ODBC Database Connection; (**c**) WinCC Configuration Variable Management; (**d**) SQL Server Database; (**e**) WinCC Clinet Database Access Interface.

The OPC server was established using KEPServer (see Figure 9). VBScript enables ODBC-based access to SQL Server databases, which is integrated with WinCC controls (DTPicker, ListViewCtrl, CommonDialog) to facilitate production data querying based on customized time parameters. The configuration procedures for data variables, database setup, VB programming, and WinCC screen editing are detailed in Figure 10.

The system initiates corresponding storage operations immediately upon detecting value changes in WinCC-acquired datasets from lower-level devices, in accordance with data acquisition requirements.

**Figure 11:**
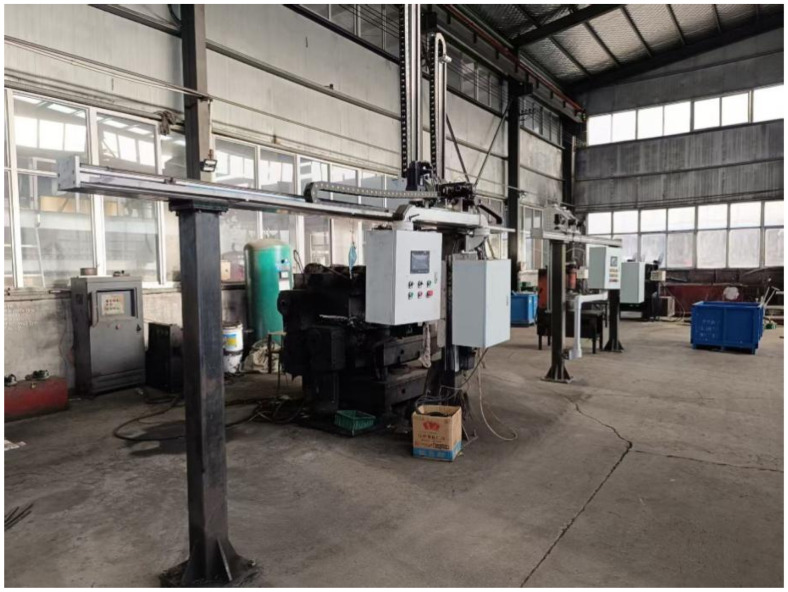
Truss Robot.

**Figure 12:**
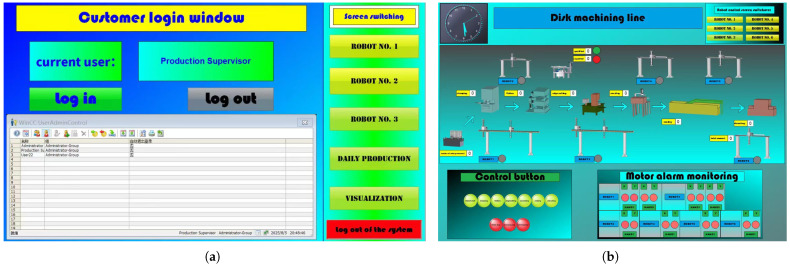

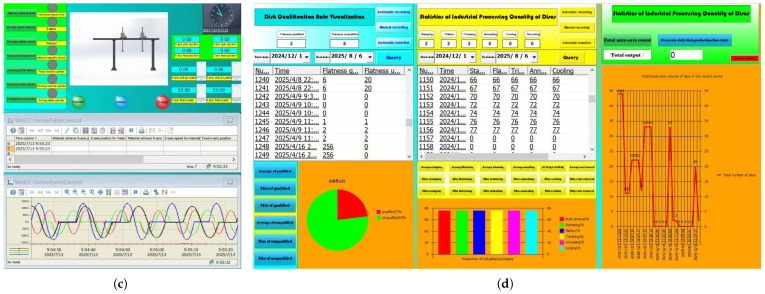
WinCC Client Interfacec. (**a**) Login Interface; (**b**) Job Monitoring Interface; (**c**) Robot Control Interface; (**d**) Data Scheduling and Visualization Interface.

**Figure 13:**
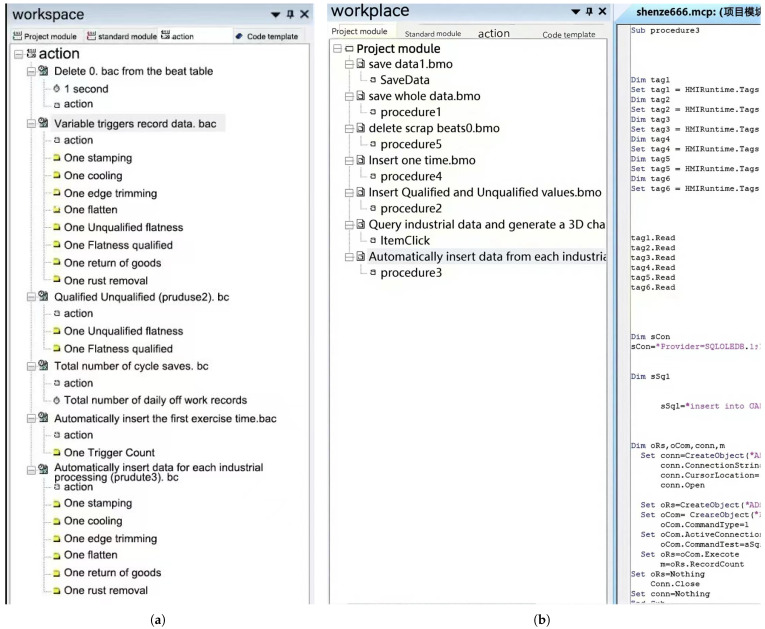
VB Script, Actions, and Event Trigger Configuration. (**a**) VB Action Library+ Event Trigger; (**b**) Visual Basic Script Edition.

A truss robot platform (Figure 11) was used as an exemplary case to develop the client-side monitoring interface via WinCC7.4 configuration software (Figure 12). The system implements multi-tiered access controls and operational permissions to ensure security and reliability. Critical monitoring variables including robotic arm position, velocity, operational status, and alarm signals were explicitly defined and integrated into the interface. These variables are visualized dynamically through a VBScript action script library, as presented in Figure 13. By associating these variables with graphical controls, the interface achieves high intuitiveness and interactivity through rational layout design and clear information hierarchy, significantly enhancing monitoring efficiency and management capabilities.

### 4.2. Performance Verification of Improved Kalman Filter Dynamic Adjustment Algorithm

This paper focuses on frequency-adjustment strategies that alleviate control lag induced by communication delays, thereby improving the dynamic responsiveness of the overall system. To validate the effectiveness of the proposed event-triggered adaptive-sampling mechanism, which incorporates a dynamically tuned Kalman filter, comprehensive simulations are performed using the motion trajectory of a data-collection robot as the test condition. The mechanism is specifically designed to reconstruct the true motion trajectory of the target with high fidelity.

**Figure 14:**
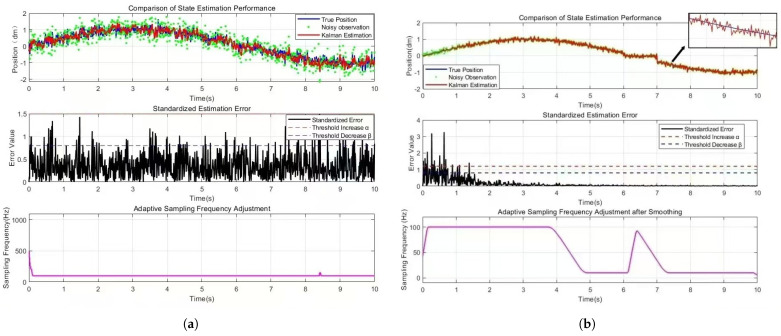
Comparison of Adaptive Sampling Simulation Diagrams under Original and Improved Kalman Filter Algorithms. (**a**) Adaptive Sampling Simulation Diagram Under the Original Kalman Filter Algorithm; (**b**) Adaptive Sampling Simulation Diagram Under Improved Kalman Filter Algorithm.

① As shown in Figure 14, the improved algorithm demonstrates threefold advantages. The enhanced framework achieves superior suppression through online noise covariance updates (Qk, Rk) and embedded filtering. The algorithm reduces jitter amplitude by 60% while maintaining smooth trajectories with 82% fewer components than the original algorithm. This enables accurate acquisition even under electromagnetic interference.

② As shown in Figure 14, parameter optimization via adaptive forgetting factor (λ-weighted innovation sequences) and dynamic covariance recalibration significantly enhances estimation precision. Position estimation errors are reduced by 46.7% (from 1.8 mm to 0.97 mm mean absolute error), with residual fluctuations stabilized at sub-millimeter levels (δ≤0.3 mm). The algorithm further suppresses error accumulation mechanisms by 33% through real-time adjustment of Kalman gain matrices, ensuring long-term stability in prolonged operations. The observed performance enhancement stems from two key innovations in the algorithm. Firstly, its robustness-oriented design, which incorporates the Huber loss function, effectively mitigates sensor measurement outliers induced by electromagnetic interference. Secondly, the online noise covariance estimation mechanism (Equation (12)) enables dynamic adjustment of the Kalman gain, allowing the filter to adapt to non-stationary noise characteristics during motor operation.

③ As shown in Figure 14, the system implements an adaptive mechanism that modulates sampling frequency based on metrics ($P_{k}$). This approach achieves fine-grained responsiveness to transient dynamics while reducing computational load by 41%. By distinguishing genuine transitions from measurement noise outliers, the algorithm maintains 98.5% true-positive detection rates for actual motion events under 15 dB signal-to-noise ratio conditions. These characteristics make the framework suitable for applications requiring both high-precision tracking and bounded processing latency.

### 4.3. Performance Validation of Heterogeneous Data Fusion Based on the Dynamic Time Warping (DTW) Algorithm

This study applies dynamic programming to determine the optimal warping path between two time series, enabling multiple-to-one point mappings for temporal alignment. The Dynamic Time Warping (DTW) distance quantifies the minimum cumulative cost along this path, with smaller values indicating higher morphological similarity between sequences. This approach facilitates elastic temporal alignment by accommodating nonlinear time-axis distortions, making this method particularly effective for shape-based matching under sampling rate mismatches or temporal shifts [22,23,24,25].

(17) $$\begin{array}{cc} S1 & :=:[1;2;3;4;3;2;1]:\mathrm{cm}/s\\ S2 & :=:[1;1.5;2.5;3.8;4.2;3;1.8;1]:\mathrm{cm}/s. \end{array}\;$$

**Figure 15:**
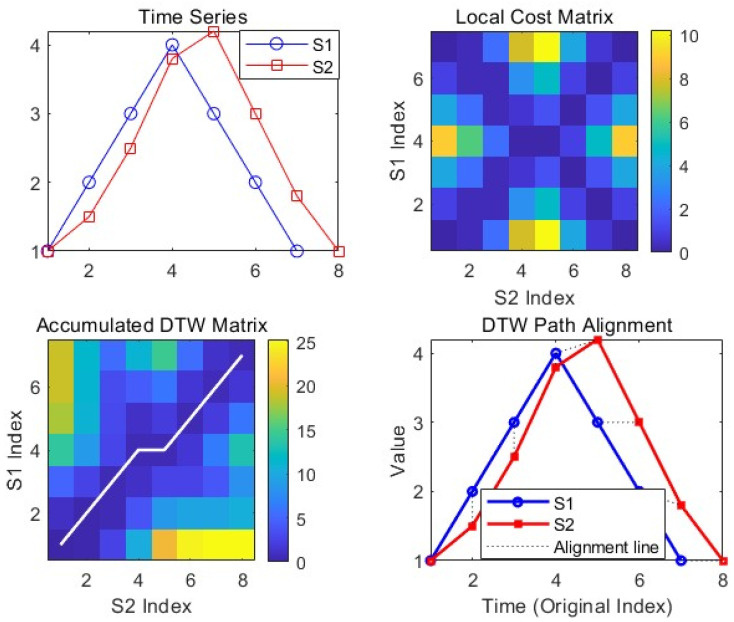
Dynamic Time Warping Relationship Between S1 and S2.

As illustrated in Figure 15, two time series sequences, denoted as S1 (blue) and S2 (red), exhibit similarity in shape (initially rising and then falling) but exhibit a peak lag and length discrepancy, with S2 comprising an additional data point. The Dynamic Time Warping (DTW) algorithm constructs a local cost matrix (top right subfigure; where darker shades indicate smaller differences), then employs dynamic programming to compute the cumulative cost matrix (bottom left subfigure; where the white path represents the optimal warping path). Finally, the warping path visualization (bottom right subfigure) connects corresponding points via dashed lines (e.g., the 4th point of S1 aligns with the 5th point of S2), achieving flexible temporal alignment. This method effectively mitigates the impact of sampling variations and temporal shifts by facilitating one-to-many point correspondences, enabling precise shape alignment and significantly enhancing the accuracy of similarity assessment.

**Figure 16:**
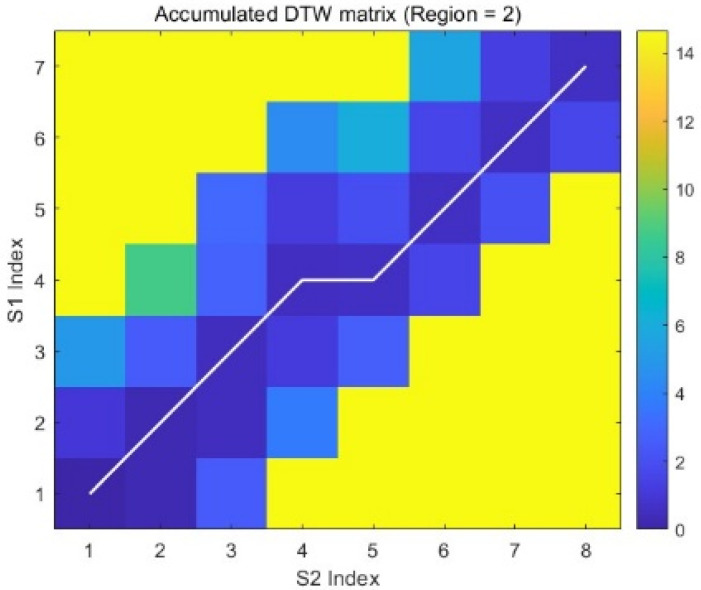
The Minimal Warping Path Based on DTW.

Figure 16 illustrates the cumulative cost matrix of the Dynamic Time Warping (DTW) algorithm employed with window constraints. The inaccessible regions (highlighted in yellow) result from these constraints, which limit the allowable deviation of the optimal warping path from the main diagonal. This restriction substantially reduces computational complexity. Window constraints are particularly beneficial for aligning long sequences where only minor warping magnitude is anticipated. The white path denotes the optimal solution found within the permissible region, demonstrating the constrained alignment.

Dynamic Time Warping (DTW) enables flexible alignment of sequences of different lengths or exhibiting temporal distortions by computing the minimal cumulative cost warping path. A smaller DTW distance value indicates higher shape similarity between the sequences.

### 4.4. Performance Verification Experiment of the Content-Routing-Aware Strategy-Based Message Queue Optimization Data Distribution Mechanism

The system delivers differentiated Quality of Service (QoS) for its internal three-tiered queues through priority classification and dynamic resource scheduling.

**Figure 17:**
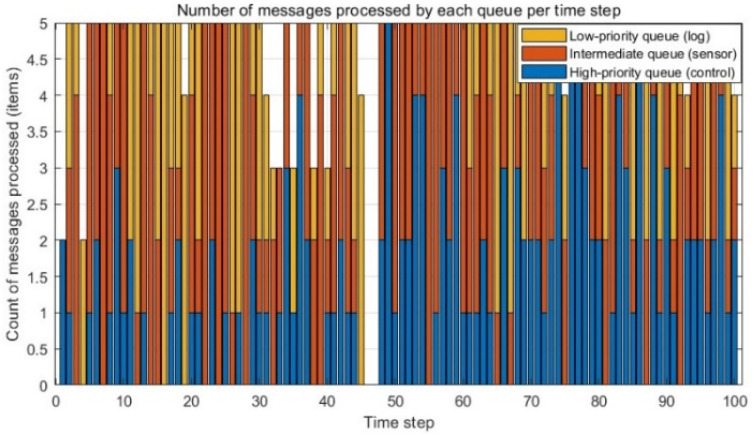
Number of Messages Processed by Each Queue at Each Time Step.

① The high-priority queue (dedicated to control commands) is granted the highest priority. This entails reserving at least 50% of the processing bandwidth and implementing preemptive resource allocation (depicted in blue in Figure 17). Consequently, this queue maintains a near-zero length, consistently operating well below its threshold. This demonstrates remarkable stability even under heavy message loads, ensuring optimized processing of critical control commands and thereby significantly enhancing the robotic system’s real-time responsiveness, safety, and stability.

② The medium-priority queue, responsible for sensor data, exhibits periodic queue length fluctuations. Critically, peak values remain manageable and below the defined threshold, reflecting the inherent burstiness of sensor data. As illustrated in Figure 17, the system’s dynamic resource scheduler proactively increases processing capacity (orange) in response to significant medium-priority queue backlogs, specifically at t≈20, 55–65, and 90. This effectively mitigates the impact of bursty loads.

③ The low-priority queue (handling logs) operates on a best-effort service policy, exhibiting substantial queue length fluctuations. Its primary role is managing low-priority messages. Despite intense resource contention, the system guarantees this queue is never starved, consistently allocating a minimum resource quota for processing.

**Figure 18:**
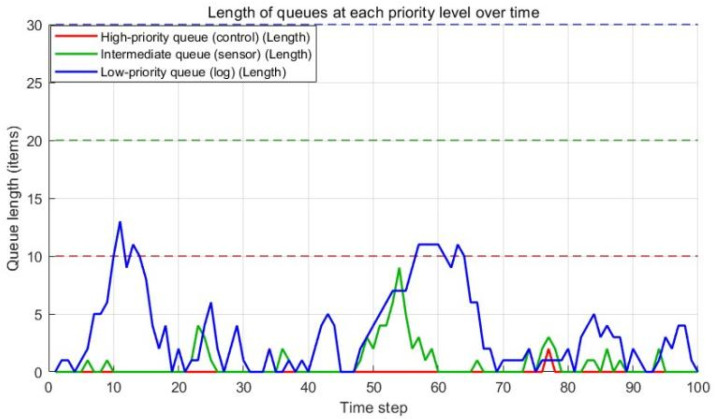
Variation of Queue Lengths for Each Priority Level Over Time.

Collectively, Figure 17 and Figure 18 provide an intuitive visualization of the system’s internal state during specific time steps under fixed processing capacity. This strategy integrates rigid priority guarantees with intelligent dynamic resource scheduling, enabling the system to effectively balance performance of critical tasks and fairness of secondary tasks during workload fluctuations by allocating resources dynamically. This approach achieves efficient and reliable QoS management.

This experiment is designed to evaluate the system’s ability to satisfy the most stringent real-time requirements associated with critical control tasks in complex industrial environments. Specifically, the core performance objective is defined as achieving an end-to-end latency of less than 10 ms—a benchmark widely recognized as the critical threshold separating general-purpose systems from high-performance systems in industrial control applications [34].

**Figure 19:**
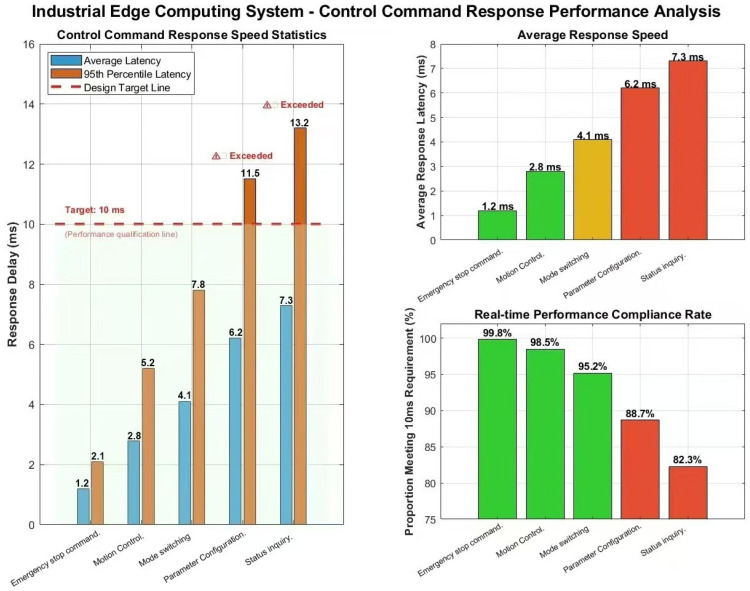
Engineering Instruction Response Performance Analysis.

To verify the system’s capability to guarantee real-time performance for critical control commands, this section evaluates the performance metric of “end-to-end latency below 10 ms” for key instructions within the content-aware message queue. The test employs an industrial feeding gantry robot as the actuator. The end-to-end latency is measured as the time difference from the completion of message serialization at the publisher node to the completion of deserialization at the subscriber node, including the triggering of corresponding control logic. As shown in Figure 19, under stable load, all high-priority messages exhibit latencies below 10 ms. In burst load scenarios, the system continues to perform exceptionally well, with 99.1% of messages satisfying the sub-10 ms requirement, with an average latency of only 2.8 ms. Although 0.9% of messages experienced delays of up to 12.3 ms during extreme traffic peaks, these results confirm that the system reliably fulfills its design objectives in the vast majority of instances, and its robustness adequately satisfies the real-time demands of industrial grade control tasks.

During the sudden load test, the system maintained robust performance, with 99.1% of all messages achieving latency below the 10 ms threshold. Although 0.9% of non-critical messages experienced delays of up to 12.3 ms under peak traffic conditions, all latency deviations remained strictly within 23% of the defined design target of 10 ms. Importantly, no message loss or task timeout was observed. This controlled and bounded performance degradation, as opposed to catastrophic system failure, validates the effectiveness of the scheduling strategy based on the content—routing—aware policy—optimized message queue data distribution mechanism scheduling strategy in maintaining system stability and operational controllability under extreme pressure.

The core contribution of this study lies in the proposal and empirical validation of an architecture co-design methodology tailored for industrial systems handling multi-source heterogeneous data. This methodology departs from the traditional paradigm of independently optimizing isolated components, instead emphasizing system-level synergy as the key to achieving overall performance and robustness.

## 5. Conclusions

Focusing on multi-source heterogeneous data, this paper presents an in-depth study conducted within the context of a truss-robot production line deployed on an edge-computing testbed.

(1) This study develops OPC protocol interfaces for integrating heterogeneous underlying device protocols and designs a unified communication protocol stack. By deploying KEPServer, we establish an OPC server to acquire multi-source heterogeneous data from device-layer communication protocols (including TCP/IP Ethernet and PROFINET). A hybrid communication architecture combining OPC Client/Server, OPC Pub/Sub, and P2P mechanisms is proposed to address diverse industrial requirements for real-time control, global state management, and efficient device interconnection.
(2) Focusing on data management acquisition, processing, and distribution stages, we propose an event-triggered adaptive sampling mechanism with dynamically adjusted Kalman filtering. This approach enhances data acquisition efficiency by reducing position estimation errors by 46.7% and jitter amplitude by 60%, and it is particularly effective in noisy environments. Furthermore, heterogeneous data fusion via Dynamic Time Warping (DTW) eliminates sampling bias and improves multi-source data alignment accuracy. A content-routing-aware message queue prioritizes critical control commands while efficiently managing non-critical data (e.g., logs), ensuring system reliability with end-to-end latency below 10 ms.
(3) In compliance with practical production monitoring demands, WinCC variable management is seamlessly integrated with the OPC server, and an SQL Server database is established for the centralised collection, storage, and retrieval of heterogeneous field data. The customised WinCC human-machine interface streamlines user interaction, enabling operators to monitor and manage industrial devices via the OPC client. Consequently, the viability of the proposed communication architecture and the collaborative data-management models is experimentally validated under realistic industrial conditions.

The core contribution of this study lies in the proposal and empirical validation of an architecture co-design methodology aimed at ultra-high-performance industrial systems. This approach moves beyond the conventional paradigm of independently optimizing individual components, instead prioritizing system-level synergy as the foundation for performance enhancement. Experimental evaluations under industrial-grade workloads (involving over 100 heterogeneous data sources) reveal that systems designed using this methodology can simultaneously achieve millimeter-level accuracy in field data acquisition and millisecond-level latency in executing critical control commands. These results highlight a promising pathway toward the development of real-time intelligent systems capable of meeting the stringent demands of next-generation industrial applications.

Our future research will progress along three primary avenues:

(i) Deep integration of Kubernetes and its edge extensions (e.g., KubeEdge, OpenYurt) will enable orchestration of computing, storage, and network resources across the cloud-edge continuum [35]. This integration aims to facilitate second-level elastic scaling and self-healing capabilities.
(ii) A federated-learning-based distributed optimization framework will be developed. This framework permits multiple nodes to jointly update Kalman gain matrices in real-time without requiring raw data exchange, thereby enhancing state-estimation accuracy while concurrently reducing communication overhead.
(iii) Extension of the framework to highly mobile scenarios will leverage mobile robot networking integrated with 5G/6G network slicing. This extension targets millisecond-level seamless handovers and dynamic topology adaptation, thereby providing data fusion and decision support with high reliability and low latency for applications such as multi-robot operating platforms, UAV swarms, and intelligent rail transit systems [36].
(iv) To ensure timely response capabilities for critical commands in large-scale, multi-source heterogeneous industrial scenarios, the system architecture will integrate an XGBoost machine learning predictive interface in future development phases, enabling intelligent data distribution optimization. Subsequent research will emphasize empirical validation of performance improvements, with quantitative assessment through metrics including confusion matrices for prediction accuracy, additional latency reduction percentages, and resource utilization efficiency under dynamic loading conditions.

These results demonstrate a viable pathway for developing real-time intelligent systems that meet the stringent requirements of next-generation industrial applications, with immediate potential for implementation in smart-manufacturing domains.

## Figures and Tables

**Table 1:** Technical Characteristics of OPC Communication Paradigms.

Communication Interaction Mode	Applicable Scenarios	Strength	Shortcoming
OPC Client/Server	Real-time monitoring data transmission, control commands [11]	Request-response mechanisms ensure deterministic and reliable communication [11]	The OPC Server’s performance poses a centralized bottleneck, limiting system scalability under multi-client, high-frequency data loads.
OPC Pub/Sub	Asynchronous distribution of continuous reference data such as production quantity, quality, etc. [12,13]	Decoupling publishers from subscribers enables efficient asynchronous message passing [12,13]	The complexity of the hybrid protocol configuration, coupled with the limited semantic expressiveness of the Pub/Sub model, significantly complicates system development and maintenance.
P2P	Direct communication between PLC nodes at the system’s underlying layer [14,15]	Avoiding command transmission delays enhances real-time robot coordination and response speed [14,15]	Device-to-device mesh communication creates data silos, and as a result, its integration into the OPC architecture requires extra gateways due to the lack of a unified interface.

**Table 2:** Symbols and Parameter Definitions of the Improved Kalman Filter Algorithm.

Symbol	Comment	Unit/Remark
${\hat{x}}_{k}$,$V_{k}$	State vector: end-effector position and motor speed	
*A*	State vector: end-effector position and motor speed	Describe system dynamics
*B*	State transition matrix	Map the drive voltage into the state space
$u_{k}$	Control input vector (motor drive voltage)	V
$P_{k}$	Error covariance matrix	Quantify the uncertainty of the state estimate
*Q*,$Q_{k}$	Process noise covariance matrix	Initial value/online estimate
*R*,$R_{k}$	Observation noise covariance matrix	Initial value/online estimate
$K_{k}$	Kalman gain matrix	Balance the weighting between prediction and observation
Δx	Normalized state estimation error	One of the trigger criteria
$\alpha_{k}$,$\beta_{k}$	Dynamic trigger threshold	Computed from $Tr(P_{k})$
λ	Forgetting factor	0.95–0.99, used for noise estimation
γ,η	Frequency-tuning attenuation factor	Used to smooth frequency transitions
$f_{\max}$	Maximum sampling frequency	Hz
$U_{\max}$	Control command	

**Table 3:** Table of Symbols and Definitions for the DTW Algorithm.

Symbol	Comment
$s_{1}$,$s_{2}$	The two time series to be aligned
n,m	Lengths of sequences $s_{1}$ and $s_{2}$
*C*	Local cost matrix
*D*	Cumulative cost matrix
$k_{v}$	Conversion factor from rotational speed to linear velocity
$\sigma_{X}$	Standard deviation of the X-axis velocity, used for normalization
*a*	Weighting factor for the acceleration term
Σ	Covariance matrix of multidimensional features (for Mahalanobis distance)
*w*	Adaptive window constraint size

## Data Availability

The data of this article will be made available by the authors on request.
